# Knowledge and Awareness Regarding Bell’s Palsy in the Al-Qassim Region, Saudi Arabia

**DOI:** 10.7759/cureus.51327

**Published:** 2023-12-30

**Authors:** Ruba M Altowayan, Samar A Alruwaysan, Seba Alraddadi, Meshal A Alanazi, Seham Alharbi, Njood M Alobaid, Lama M Aldakhil, Abdulaziz F Almohaimeed, Tameem A Alhomaid

**Affiliations:** 1 Unaizah College of Medicine and Medical Sciences, Qassim University, Qassim, SAU; 2 College of Medicine, Jouf University, Sakakah, SAU; 3 Department of Public Health, Department of Medicine and Surgery, King Fahad Specialist Hospital, Buraydah, SAU; 4 Department of Family Medicine, Qassim Health Cluster, Buraydah, SAU

**Keywords:** cranial nerve palsy, facial paralysis, facial palsy, bell’s palsy, awareness, knowledge

## Abstract

Introduction*:* Bell's palsy, characterized by acute onset unilateral facial weakness, is caused by the paralysis of the seventh cranial nerve, which controls the muscles of the face. This condition can result in functional disabilities, and early detection and management are crucial for quick recovery. Awareness was found to be one of the factors associated with early detection and interventions. Therefore, this study aimed to assess the awareness of the population of Al-Qassim, Saudi Arabia, regarding Bell’s palsy.

Methods: We conducted a cross-sectional study on 1,198 participants in Al-Qassim, Saudi Arabia, between May and July 2023. We used a self-administered online questionnaire inquiring about knowledge and awareness of Bell’s palsy. We performed descriptive and correlation analyses, and a p-value of less than 0.05 indicated a statistical significance.

Results: The mean (± SD) knowledge score was 7.02 ± 2.03 out of a total of 13 points. Almost a third of participants (n=353, 29.5%) expressed uncertainty about the causes of Bell's palsy, with 346 (28.9%) and 107 (8.9%) attributing it to idiopathic factors and viral infections, respectively. Most participants (n=520, 43.4%) believed both genders were equally affected, while 563 (46.9%) correctly identified cranial nerve 7 as the affected nerve. Treatment awareness varied, with 629 (58.2%) acknowledging physiotherapy and (n=777, 64.9%) acknowledging traditional medicine. Interestingly, only 111 (9.3%) thought that Bell's palsy was permanent, most participants (n=1023, 85.4%) recognized Bell's palsy as treatable, and 1,105 (92.2%) correctly perceived it as non-contagious. There were significant correlations between awareness and age (p<0.001), gender (p<0.001), marital status (p<0.001), occupation (p<0.001), information source (p<0.001), nationality (p=0.009), and education levels (p<0.031). Addressing these gaps and demographic nuances through targeted educational campaigns is crucial for enhancing overall awareness of Bell's palsy.

Conclusion: These findings indicate suboptimal awareness among participants in general, poor knowledge about causes and clinical manifestation, and a relatively better awareness of treatments. We recommend further studies exploring awareness and associated factors.

## Introduction

Bell's palsy is a transient condition characterized by sudden unilateral facial weakness or paralysis resulting from damage to the 7^th^ cranial nerve [1]. Bell’s palsy results in facial discomfort, deformity, psychological damage, and facial muscle weakness, especially on one side of the face [2]. Lower motor neuron facial palsy is most commonly caused by Bell's palsy, an acute peripheral facial neuropathy that can get worse within 48 hours from onset [3]. In Bell's palsy patients, the muscles that control facial expressions become flaccid and paralyzed in a unilateral (rarely bilateral) fashion [4]. Speech, chewing, sucking, swallowing, and lip holding may become hyperacusic and hypoesthetic, and there may be functional deficiencies. It may also be accompanied by problems with taste, salivation, and tearing [4]. The clinical presentation is typically the disappearance of facial creases and nasolabial folds, the unfurrowing of the brow, and the drooping of the corner of the mouth. The eyelids are unable to close, and the lower lid sags; when closed, the eye rolls upward (Bell's phenomenon) [5]. The Bell's phenomenon, also known as the palpebral-oculogyric reflex, is the upward movement of the eyeballs when the eyelids are forcefully closed [5,6].

The anatomical structure of the facial nerve, particularly its mixed neural profile containing motor, sensory, and parasympathetic fibers, can explain this clinical presentation [1]. Several risk factors for Bell's palsy have been identified thus far. Patients who are pregnant, have severe preeclampsia, are obese, have high blood pressure including chronic hypertension, and diabetes, or have upper respiratory diseases/infections are more likely to develop Bell's palsy [7].

Most (60-75%) cases of Bell's palsy are thought to be idiopathic [1]. Other less common causes include head and neck tumors, infections, neurologic disorders, neoplasia, trauma, and congenital instances [8]. Bell's palsy was found to have an annual incidence rate of 11-50 cases per 100,000 people, with a median age of 40 years worldwide [9]. Children experience it less frequently, and men and women are equally affected, so there is no difference in the prevalence between the sexes [10]. However, another study conducted in the Qurayyat region showed that Bell’s palsy was more prevalent among males (61%) [11]. The majority of people with Bell's palsy recover without any treatment, giving the condition a good prognosis. However, 30% of patients may experience facial muscular weakness, deformity, psychological trauma, and facial pain, which can lead to poor prognosis [10].

A study conducted in the Western region of Saudi Arabia showed that 85% of participants from Makkah, Jeddah, and Taif cities were aware of Bell's palsy, compared to 21% who were unaware of Bell’s palsy [10]. Another previous study contrasted this and indicated that the general Saudi population had poor awareness of Bell's palsy, particularly its signs and symptoms. However, a better understanding of the etiology and treatment has been documented [12]. These findings highlight the need to raise awareness about Bell’s palsy among Saudi nationals to improve the treatment outcomes. One study showed that 18.7% of patients preferred traditional herbal medicine for Bell’s palsy, which is ineffective [11]. Raising awareness could help affected patients seek appropriate care. Since there is a lack of studies evaluating knowledge and awareness regarding Bell’s palsy in Saudi Arabian regions, particularly the Qassim region, our study aimed to assess awareness of the population of Al-Qassim, Saudi Arabia, regarding Bell’s palsy.

## Materials and methods

Study design: We conducted a cross-sectional, questionnaire-based study in all Al-Qassim regions between May and July 2023, and we targeted adult residents of the Al-Qassim region.

Sampling: The sample size was estimated by an online sample size calculator (Raosoft, http://www.raosoft.com/samplesize.html) and by using a margin of error of 5% and a confidence interval of 95%, assuming an average response for most of the questions of 50%. According to the population of 1,016,756 in Al-Qassim, Saudi Arabia, the required minimum sample size was 385. Participants were selected using a simple random sampling technique.

Data collection methods: We used a valid pre-tested structured online questionnaire adapted from a previously published study by Alherabi et al. [10]. The questionnaire included demographic information. The questionnaire also included questions on demographic information, knowledge, and awareness of Bell's palsy. A panel of three experts reviewed the questionnaire items to determine their validity during the validity assessment. The assessment was completed independently at first, and then disputed items were thoroughly discussed until a consensus was reached. All suggested modifications were implemented to improve the validity of the questionnaire until the final format used in the current study was obtained. Cronbach's alpha of 0.73 showed its reliability. The questionnaire was further pilot-tested on 25 participants, and the results were used to improve clarity and easy understanding of the questions. The questionnaire was translated into Arabic using the forward-backward method to ensure all participants' understanding.

The questionnaire was distributed online as the Google Form link via WhatsApp, Telegram, Twitter, and other social media platforms. The Google Form link also contained a letter inviting participants to voluntarily participate, with details on the purpose of the study and informed consent.

Data analysis: Data were analyzed using Statistical Product and Service Solutions (SPSS, version 26) software (IBM SPSS Statistics for Windows, Armonk, NY). We performed descriptive statistics, and numeric data were presented as mean ± SD or as median and range according to the type of distribution of each variable. For categorical variables, frequency and percentages were used. Crosstabulation was used to assess the distribution of awareness levels based on the participant's personal data and information sources. Pearson's chi-squared test and exact probability test for small frequency distributions were used to test relationships. A p-value of <0.05 was considered statistically significant.

Ethical considerations: Participation was voluntary after obtaining informed consent before filling out the questionnaire. The confidentiality was ensured by the anonymity of the questionnaire. This study was approved by the Regional Ethical Committee of Qassim Region, Saudi Arabia.

## Results

**Figure 1:**
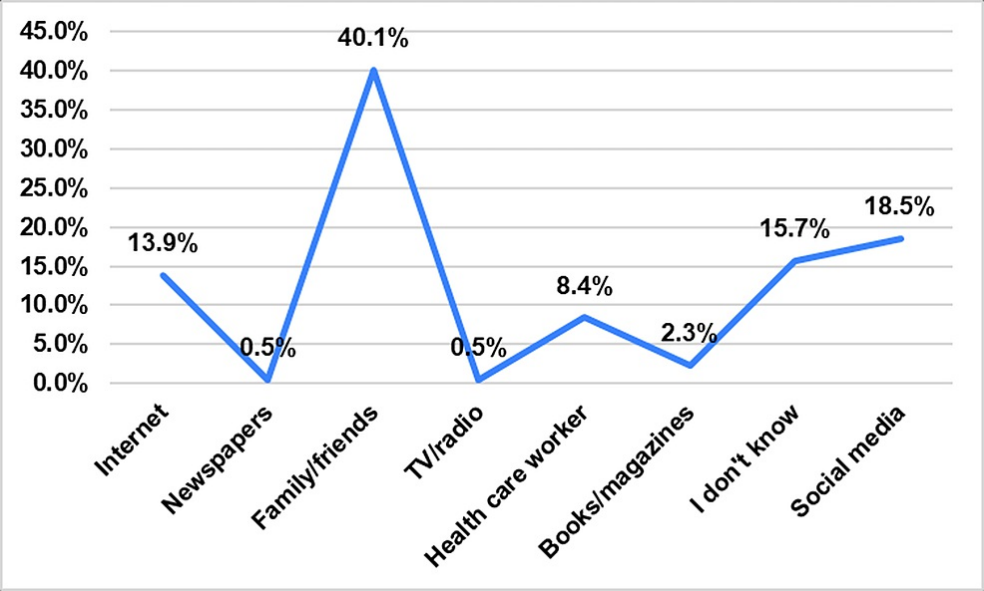
Source of information regarding Bell’s palsy

As shown in Table 1, there was a total of 1,198 participants in this study who successfully completed the questionnaires. The majority (n=493; 41.2%) were between 36 and 50 years old. Most participants were female (n=981; 81.9%) and married (n=734; 61.3%). Occupationally, participants are almost evenly split between the employed (n=532; 44.4%) and the non-employed (n=666; 55.6%). Over two-thirds held a university degree (n=817; 68.2%). Nearly all participants (n=1,192; 99.5%) were Saudi nationals, and 92.6% (n=1,109) of the participants were Al-Qassim residents. Most participants receive information on Bell’s palsy from family and friends (n=480; 40.1%), followed by social media (n=222; 18.5%). However, 188 (15.7%) did not know their source of information on Bell’s palsy (Figure 1).

**Table 1:** Sociodemographic characteristics data of the study participants

Personal data		N	%
Age groups	<20	135	11.3
	20-35	416	34.7
	36-50	493	41.2
	>50	154	12.9
Gender	Female	981	81.9
	Male	217	18.1
Marital status	Widowed	21	1.8
	Single	403	33.6
	Married	734	61.3
	Divorced	40	3.3
Occupation	No	666	55.6
	Yes	532	44.4
Level of education	Primary school	20	1.7
	Middle School	37	3.1
	Secondary school	253	21.1
	University	817	68.2
	Post-graduate	71	5.9
City of residence	Qassim	1109	92.6
	Outside Qassim region	89	7.4
Nationality	Saudi	1192	99.5
	Non-Saudi	6	0.5
Source of information regarding facial palsy	Internet	167	13.9
	Newspapers	6	0.5
	Family/friends	480	40.1
	TV/radio	6	0.5
	Health care worker	101	8.4
	Books/magazines	28	2.3
	I don't know	188	15.7
	Social media	222	18.5

**Figure 2:**
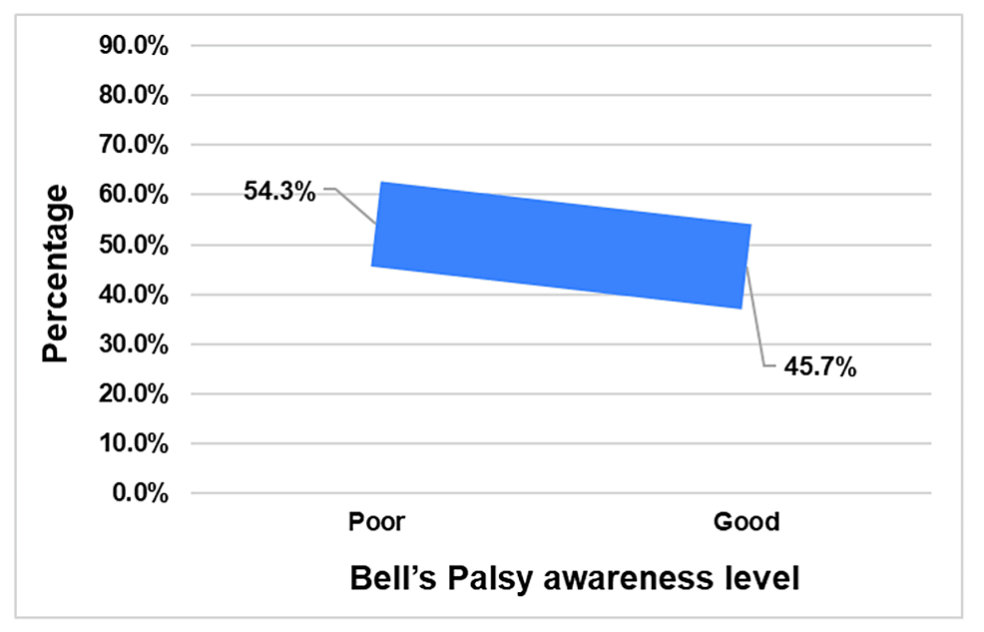
Overall awareness regarding Bell’s palsy among the general population in the Al-Qassim region, Saudi Arabia

On the other hand, the study's overall assessment of awareness regarding Bell's palsy indicated that most participants (n= 650; 54.3%) exhibited poor knowledge, and the mean (± SD) knowledge score was 7.02 ± 2.03 out of a total of 13 points (Figure 2).

Table 2 shows Bell’s palsy awareness among study participants. A notable proportion of participants expressed uncertainty about the causes of Bell's palsy (n=353; 29.5%), and some were aware of idiopathic factors (n=346; 28.9%) and viral infections (n=107; 8.9%) as causes. Awareness about the affected demographic varies, with the majority suggesting an equal impact on both genders (n=520; 43.4%). The majority correctly identified cranial nerve 7 as the nerve affected in facial palsy (n=562; 46.9%); nevertheless, 525 (43.8%) were unsure. Less than 10% believed that Bell's palsy is a lifelong condition (n=111; 9.3%).

**Table 2:** Bell’s palsy awareness among study participants

Bell’s palsy awareness items		N	%
What causes Bell’s palsy?	Stroke	205	17.1
	shock	187	15.6
	Viral infection	107	8.9
	I don't know	353	29.5
	Idiopathic	346	28.9
Which group does Bell’s palsy affect more?	men	118	9.8
	Women	187	15.6
	Equally affected	520	43.4
	I don't know	373	31.1
Which nerve is affected in facial nerve palsy?	Other	24	2.0
	Cranial nerve 1	14	1.2
	Cranial nerve 11	19	1.6
	Cranial nerve 5	54	4.5
	Cranial nerve 7	562	46.9
	I don't know	525	43.8
Bell’s palsy is generally a self-limiting disease	False	609	50.8
	True	251	21.0
For how long does Bell’s palsy persist in an affected individual?	It is a lifelong disease	111	9.3
	Weeks to months	601	50.2
	Days to a week	137	11.4
	Hours to days	29	2.4
	I don't know	320	26.7
Do you think Bell’s palsy is a treatable disease?	No	38	3.2
	I don't know	137	11.4
	Yes	1023	85.4
What is the treatment of Bell’s palsy?	Steroids	58	4.8
	Spiritual treatment	3	0.3
	Herbal medicine	20	1.7
	Physiotherapy	697	58.2
	I don't know	298	24.9
	Pain killers	22	1.8
	Antibiotics	57	4.8
	Antivirals	43	3.6
Do you think traditional medicine has a role in treating Bell’s palsy?	No	421	35.1
	Yes	777	64.9
Have you ever been diagnosed with facial palsy?	No	1131	94.4
	Yes	67	5.6
Have you ever heard about someone you know who had been diagnosed with facial palsy?	No	320	26.7
	Yes	878	73.3
What is your relation to the person who had been diagnosed with facial palsy?	Other	256	21.4
	neighbor	52	4.3
	A friend	220	18.4
	Family	512	42.7
	Not applicable	158	13.2
Do you think Bell’s palsy is a contagious disease?	No	1105	92.2
	I don't know	74	6.2
	Yes	19	1.6

Concerning treatment awareness, physiotherapy was acknowledged by a majority (n=697; 58.2%), and 777 (64.9%) thought traditional medicine was one of the treatment options. The majority of participants were aware that Bell's palsy is a treatable condition (n=1023; 85.4%), and 878 (73.3%) had heard about someone diagnosed with facial palsy, primarily within their family (n=512; 42.7%). However, slightly over a quarter (n=320; 26.7%) remained unaware of anyone with this diagnosis. Encouragingly, the majority accurately perceive Bell's palsy as non-contagious (1105; 92.2%).

Table 3 shows the correlation between participants’ awareness regarding Bell’s palsy band, their demographic data, and their source of information. There was a statistically significant difference in awareness levels, according to age (p<0.001), with good awareness levels more prevalent among those aged 36-50 (262/493; 53.1%). There was also a significant correlation between awareness and gender (p<0.001), with good awareness among females (475/981; 48.4%) compared to males (73/217; 33.6%). The marital status was also correlated with awareness of Bell’s palsy (p<0.001), with good awareness more observed among married participants (386/734; 52.6%). Employment, nationality, and education level were also significantly correlated with participants' awareness (p<0.001, p=0.009, and p=0.031, respectively), with good awareness more observed among employed (284/532; 53.4%), those with primary education levels (13/20; 65%), and non-Saudi nationals (n=6; 100%). The source of information about Bell’s palsy was also correlated with awareness, with good awareness more prevalent among participants obtaining information from TV/radio (5/6; 83.3%) (p<0.001).

**Table 3:** Distribution of study participants’ awareness regarding Bell’s palsy by their personal data and source of information BP: Bell's palsy. a: Pearson X2 test. b: Exact probability test. * P < 0.05 (significant). ** P < 0.01 (significant)

BP awareness items		Awareness level				P-value
		Poor		Good		
		N	%	N	%	
Age groups ^a^	<20	95	70.4%	40	29.6%	<0.001**
	20-35	247	59.4%	169	40.6%	
	36-50	231	46.9%	262	53.1%	
	>50	77	50.0%	77	50.0%	
Gender ^a^	Female	506	51.6%	475	48.4%	<0.001**
	Male	144	66.4%	73	33.6%	
Marital status ^a^	Widowed	12	57.1%	9	42.9%	<0.001**
	Single	269	66.7%	134	33.3%	
	Married	348	47.4%	386	52.6%	
	Divorced	21	52.5%	19	47.5%	
Occupation ^a^	No	402	60.4%	264	39.6%	<0.001**
	Yes	248	46.6%	284	53.4%	
Level of education ^a^	Primary school	7	35.0%	13	65.0%	0.031*
	Middle School	26	70.3%	11	29.7%	
	Secondary school	131	51.8%	122	48.2%	
	University	440	53.9%	377	46.1%	
	Post-geadute	46	46.8%	25	35.2%	
City of residence ^a^	Qassim	609	54.9%	500	45.1%	0.107
	Outside Qassim region	41	46.1%	48	53.9%	
Nationality ^b^	Saudi	650	54.5%	542	45.5%	0.009**
	Non-Saudi	0	0.0%	6	100.0%	
Source of information regarding facial palsy ^b^	Internet	93	55.7%	74	44.3%	<0.001**
	Newspapers	4	66.7%	2	33.3%	
	Family/friends	234	48.8%	246	51.3%	
	TV/radio	1	16.7%	5	83.3%	
	Health care worker	40	39.6%	61	60.4%	
	Books/magazines	15	53.6%	13	46.4%	
	I don't know	146	77.7%	42	22.3%	
	Social media	117	52.7%	105	47.3%	

## Discussion

Bell's palsy is a neurological disorder of facial muscles, resulting in temporary paralysis [3]. People need to be well-informed about this condition, as early recognition and appropriate treatment can significantly impact recovery. Our study has investigated awareness levels of Bell's palsy among Al-Qassim residents, revealing both strengths and areas for improvement in public knowledge.

This study's findings showed that most participants acquire information about Bell's palsy from family and friends (40.1%). Social media also plays a role, with 18.5% receiving information from social media platforms. Our findings align with previous studies [13] showing family and friends as the source of information. Studies also highlighted the significance of social media in spreading Bell’s palsy information [14,15]. It was found that the use of reels (p<0.001) and the profile host by healthcare providers, interacting with their followers by liking (p<0.001), and replying (p<0.001) to comments increased engagement significantly in raising awareness about Bell’s palsy [14]. Our study showed that 15.7% were unsure about the source of their knowledge. This implies that more structured and reliable information dissemination channels are required to ensure accurate and consistent awareness.

The study's assessment of overall awareness revealed that a majority of participants (54.3%) exhibited poor knowledge about Bell's palsy, with a mean knowledge score of 7.02 ± 2.03 out of a possible 13 points, emphasizing the importance of improved education and awareness campaigns. This score is higher than the reported scores in Makkah, Jeddah, and Taif regions (5.6 ± 2.3) and nationwide (6.65 ± 2.3) [10,12]. These findings in our study and those previous studies indicate a knowledge gap that needs to be addressed to improve understanding and outcomes for people with Bell's palsy in Saudi Arabia.

Almost a third of participants expressed uncertainty about the causes of Bell's palsy (29.5%), indicating the need for clearer information dissemination. Close percentages (26.1%) were also reported in Western regions of Saudi Arabia [10]. A previous study conducted nationwide in Saudi Arabia reported that viral (52.9%) and idiopathic (51.7%) were major causes of Bell’s palsy [12], indicating higher awareness of causes than our study’s participants. Our study showed that idiopathic factors and viral infections were identified by 28.9% and 8.9% of participants, respectively. We also found awareness variations about the affected demographic, with 43.4% of participants suggesting an equal impact on both genders. This is consistent with ambiguous evidence from the literature. Some studies have established that Bell’s palsy significantly affects more male than female patients [11,16]. However, other studies contrasted them by showing that female patients are more affected [17]. Another study conducted on children showed that females were more affected [18]. This underscores the importance of further studies on the impact of age on the gender distribution of Bell’s palsy prevalence.

While nearly half of the participants correctly identified cranial nerve 7 as the nerve affected in facial palsy (46.9%), 43.8% were unsure. Similar findings were reported in another study [10]. Since the general population is more likely to be unaware of the anatomy and pathology of Bell’s palsy, campaigns providing clear information about the anatomical aspects of Bell's palsy could contribute to improved understanding among the general population. Less than 10% of participants (9.3%) thought that Bell's palsy was permanent. This misconception might influence individuals' attitudes toward seeking timely medical attention and adhering to prescribed treatments. Public health professionals and healthcare providers should conduct education campaigns to correct misconceptions and promote proactive healthcare behavior. Encouragingly, 85.4% of participants were aware that Bell's palsy is treatable, highlighting the importance of disseminating information about available treatment options. These findings align with a previous study, showing a better understanding of the etiology and treatment of Bell's palsy, with some misconceptions [12]. Physiotherapy to manage Bell's palsy was found to be effective [19]. Our study also found that the majority of participants (58.2%) recognized physiotherapy as a viable treatment option. However, traditional medicine was viewed as another treatment option by 64.9%, indicating a need for spreading more information on evidence-based treatments. Though the use of acupuncture and herbs was found to be effective in treating Bell’s palsy, research is not yet conclusive, and more studies should be conducted [20].

Aligning with other previous studies [21,22], most participants had heard about someone diagnosed with facial palsy, primarily within their family. This may be attributed to the reported association between recurrent Bell’s palsy and family history due to possible hereditary contribution [22]. Encouragingly, the vast majority of participants perceived Bell's palsy as non-contagious (92.2%), which is a correct perception aligning with the scientific evidence showing that Bell’s palsy is not contagious [7,17,23]. This good perception among participants is crucial for dispelling unnecessary fears and fostering a more informed community, leading to early seeking of care and optimal outcomes.

Our findings showed that age, gender, marital status, employment, nationality, education level, and the source of information about Bell's palsy were all significantly correlated with participants' awareness. Previous studies align with our findings by showing similar factors associated with awareness [10,12,14]. Alherabi et al. [10] found that awareness increased with age; female participants had better awareness, and those who read books and magazines/book readers were more aware. Our findings are similar in terms of awareness correlation with age and gender. However, they showed that TV/radio, and being employed were correlated with high awareness levels.

There are limitations to consider for this study, including the possibility of sampling bias, and response biases due to online design, the reliance on self-reported data with the risk of recall bias, and the use of a cross-sectional design, which limits the examination of awareness trends over time. This study was centered in a single region, limiting its generalizability beyond the Al-Qassim region, and the questionnaire design may not capture the breadth of participants' understanding. Furthermore, the study cannot prove a cause-effect relationship between demographic factors and awareness levels.

## Conclusions

The findings of this study showed suboptimal awareness of the causes, signs, and symptoms of Bell’s palsy. However, the awareness of treatment was relatively better, though there was room for improvement. The findings highlight the need for targeted awareness campaigns to address gaps in knowledge and awareness about Bell's palsy. Tailored efforts should focus on providing clear and accurate information regarding causes, demographics, anatomical aspects, and available treatments, as well as busting myths. Moreover, by targeting specific demographic factors identified in the study, such campaigns can enhance overall awareness and contribute to improved outcomes for patients affected by Bell's palsy. Finally, we recommend further studies exploring factors associated with knowledge and awareness to inform targeted measures and strategies.

## References

[1] Warner MJ, Hutchison J, Varacallo M (2023). Bell palsy. StatPearls.

[2] Newadkar UR, Chaudhari L, Khalekar YK (2016). Facial palsy, a disorder belonging to influential neurological dynasty: review of literature. N Am J Med Sci.

[3] Eviston TJ, Croxson GR, Kennedy PG, Hadlock T, Krishnan AV (2015). Bell's palsy: aetiology, clinical features and multidisciplinary care. J Neurol Neurosurg Psychiatry.

[4] Cappeli AJ, Nunes HRDC, Gameiro MDOO, Bazan R, Luvizutto GJ (2020). Main prognostic factors and physical therapy modalities associated with functional recovery in patients with peripheral facial paralysis. Fisioter Pesqui.

[5] McFarlin A, Peckler B (2008). An unusual presentation of Bell's palsy: a case report and review of literature. J Emerg Trauma Shock.

[6] Nowak-Gospodarowicz I, Rękas M (2021). Predicting factors influencing visual function of the eye in patients with unresolved facial nerve palsy after upper eyelid gold weight loading. J Clin Med.

[7] Baugh RF, Basura GJ, Ishii LE (2013). Clinical practice guideline: Bell's palsy. Otolaryngol Head Neck Surg.

[8] Gilden DH (2004). Bell's palsy. N Engl J Med.

[9] Zohrevandi B, Monsef Kasmaee V, Asadi P, Tajik H (2014). Report of 121 cases of Bell’s palsy referred to the emergency department. Emerg (Tehran).

[10] Alherabi A, Al-Khatib T, Sheffah FirasRA, Alturki Y, Alqurashi H, Alhassoun A, Alqurashi O (2021). Knowledge and awareness regarding Bell’s palsy among the general population in the western region of Saudi Arabia. J Health Sci.

[11] Alanazi F, Kashoo FZ, Alduhishy A, Aldaihan M, Ahmad F, Alanazi A (2022). Incidence rate, risk factors, and management of Bell's palsy in the Qurayyat region of Saudi Arabia. PeerJ.

[12] Alamrani S, Rummani S, Khamdan Z (2020). Awareness of general adult population of Saudi Arabia toward Bells palsy. IJMDC.

[13] Norris JH, Longmire NM, Kilcoyne S, Johnson D, Fitzpatrick R, Klassen AF (2019). Exploring patient experience of facial nerve palsy to inform the development of a prom. Plast Reconstr Surg Glob Open.

[14] Knoedler L, Knoedler S, Chartier C (2023). The rise of facial palsy on social media over the last 5 years. J Craniofac Surg.

[15] Walker H, Reece MK, Kadakia SP (2023). Discussion on the rise of facial palsy on social media over the last 5 years. J Craniofac Surg.

[16] Jeong J, Yoon SR, Lim H, Oh J, Choi HS (2021). Risk factors for Bell's palsy based on the Korean National Health Insurance Service National Sample Cohort data. Sci Rep.

[17] Varga E, Battamir U, Szegedi I, Hudák L, Kovács N, Nagy AC (2023). Seasonal patterns in the epidemiology of Bell's palsy in Hungary. Front Neurol.

[18] Rowhani-Rahbar A, Baxter R, Rasgon B, Ray P, Black S, Klein JO, Klein NP (2012). Epidemiologic and clinical features of Bell's palsy among children in Northern California. Neuroepidemiology.

[19] Ferreira M, Marques EE, Duarte JA, Santos PC (2015). Physical therapy with drug treatment in Bell palsy: a focused review. Am J Phys Med Rehabil.

[20] Yu G, Luo S, Zhu C (2023). Global trends and performances of acupuncture therapy on Bell’s palsy from 2000 to 2023: a bibliometric analysis. J Pain Res.

[21] Rowhani-Rahbar A, Klein NP, Lewis N (2012). Immunization and Bell's palsy in children: a case-centered analysis. Am J Epidemiol.

[22] Qin D, Ouyang Z, Luo W (2009). Familial recurrent Bell's palsy. Neurol India.

[23] Greco A, Gallo A, Fusconi M, Marinelli C, Macri GF, de Vincentiis M (2012). Bell's palsy and autoimmunity. Autoimmun Rev.

